# Brain metabolic changes associated with post-stroke pathological laughing and crying: an ^18^F-FDG-PET study in pontine stroke

**DOI:** 10.3389/fneur.2025.1641045

**Published:** 2025-07-21

**Authors:** Soojin Choi, Dae Hyun Kim, Won Jun Kang, Yong Wook Kim

**Affiliations:** ^1^Department of Physical Medicine and Rehabilitation, Hanyang University Medical Center, Seoul, Republic of Korea; ^2^Department of Medicine, Graduate School, Yonsei University College of Medicine, Seoul, Republic of Korea; ^3^Department of Physical Medicine and Rehabilitation, Samsung Medical Center, Sungkyunkwan University School of Medicine, Seoul, Republic of Korea; ^4^Department of Nuclear Medicine, Severance Hospital, Yonsei University College of Medicine, Seoul, Republic of Korea; ^5^Department and Research Institute of Rehabilitation Medicine, Yonsei University College of Medicine, Seoul, Republic of Korea

**Keywords:** pathological laughing and crying, pontine stroke, brain metabolism, emotional regulation, PLACS

## Abstract

**Background:**

Pathological laughing and crying (PLC) is characterized by sudden, uncontrollable, and inappropriate episodes of laughter or crying. While previous studies have identified PLC-associated structural lesions, the underlying metabolic alterations in these patients remain unclear.

**Objective:**

We aimed to investigate cerebral metabolic alterations in patients with PLC following pontine stroke using ^18^F-fluorodeoxyglucose-positron emission tomography imaging.

**Methods:**

In this retrospective study, we included 49 patients with pontine stroke admitted to a tertiary inpatient rehabilitation hospital between January 2011 and December 2021. Patients were classified into PLC (*n* = 20) and non-PLC (*n* = 29) groups. ^18^F-fluorodeoxyglucose-positron emission tomography images obtained within 14 days of admission were analyzed using the SPM 12 software. Voxel-wise two-sample t-tests were performed to compare brain metabolism between the two groups (*P*_family-wise error-corrected_ < 0.05). Multiple regression analysis was conducted to identify brain regions significantly associated with PLC severity, adjusting for age and stroke lesion volume.

**Results:**

Compared with that of the non-PLC group, the PLC group exhibited significant hypometabolism in the right superior frontal gyrus (*P*_family-wise error-corrected_ < 0.05). Multiple regression analysis revealed that decreased metabolism in the right inferior and middle temporal gyri was significantly correlated with higher Pathological Laughter and Crying Scale scores, indicating greater PLC severity. No brain regions showed positive correlations with the Pathological Laughter and Crying Scale scores.

**Conclusion:**

Our findings reveal that PLC following pontine stroke is associated with distinct patterns of hypometabolism, particularly in the right superior frontal gyrus and the right inferior and middle temporal gyri. These regions may contribute to the regulation of emotional expression and provide insights into the neural mechanisms underlying PLC.

## 1 Introduction

Pathological laughing and crying (PLC) is characterized by sudden, uncontrollable, and inappropriate episodes of laughter or crying. These episodes are typically brief, intense, and occur suddenly and frequently (1, 2). PLC is a disorder of emotional expression rather than a primary emotional disturbance. It involves a disturbance in the expression of emotions, and a pathologically lowered threshold for emotional responses, whereby even non-emotional stimuli or trivial emotional triggers can inappropriately provoke these episodes. Ultimately, PLC can substantially impair a patient’s emotional wellbeing, sleep quality, social abilities, and overall quality of life, often disrupting daily activities. Moreover, PLC can cause considerable long-term distress, not only for affected individuals but also for their families.

PLC is believed to result from neurological disorders; however, its exact pathophysiology remains unclear. It has been reported in patients with various neurological disorders, including stroke, multiple sclerosis, Parkinson’s disease, amyotrophic lateral sclerosis, and other neurodegenerative disorders (1, 3). Among patients with stroke, PLC is estimated to develop in approximately 11–34%, with the highest incidence within the 1st year post-stroke (1). The prevalence is notably higher during the acute phase of stroke and among hospitalized patients. Despite its clinical significance, PLC often remains under-recognized and misinterpreted by both family members and clinicians, resulting in undertreatment (4–7).

Although many cases of PLC have been reported following pontine stroke (8, 9), the condition is thought to result not from a single focal legion but from disruption of a distributed neural network. In particular, dysfunction within the frontal cortex, cortico-pontine pathways, and cerebellar circuits—collectively referred to as cortico-pontine-cerebellar network—is believed to play a central role in the development of PLC (10–12). This network is involved in the motor modulation of affective emotional expression, and its disruption may contribute to the emotional dysregulation characteristic of PLC (10–12). Additionally, the cerebellum is increasingly recognized as part of an integrated neural system responsible for cognitive and affective processing. Emotional lability, including that seen in PLC, has been reported following cerebellar disorders, further supporting its involvement in affective regulation (13–15).

Several previous studies have investigated PLC using magnetic resonance imaging (MRI)-based structural analyses, connectivity studies, and lesion-network-symptom mapping (using functional MRI) (8, 10, 11, 16, 17); however, no study to date has examined brain glucose metabolism using ^18^F-fluorodeoxyglucose (^18^F-FDG)-positron emission tomography (PET) in patients with PLC following pontine stroke. FDG-PET allows for the assessment of metabolic activity in functionally connected brain regions and has been widely used to investigate brain metabolism in many neurodegenerative diseases, cognitive disorders, and strokes (18–20). Accordingly, we selected this modality to explore PLC-associated regions. Since PLC occurs not only in stroke but also in various neurodegenerative and demyelinating conditions, FDG-PET was considered a suitable approach to assess potential functional relationships.

In this study, we aimed to evaluate the functionally related brain regions and their involvement in PLC in patients with pontine stroke by analyzing glucose metabolic differences using ^18^F-FDG-PET imaging. Furthermore, we investigated the anatomical regions associated with PLC severity, as measured using the Pathological Laughter and Crying Scale (PLACS). Through this approach, we aimed to clarify the neural correlates and underlying mechanisms of PLC.

## 2 Materials and methods

### 2.1 Procedures

In this retrospective study, we could not estimate effect size based on prior ^18^F-FDG-PET studies using the same cohort, as no such studies currently exist. Hence, it was conducted as a retrospective case–control study to compare brain glucose metabolism between patients with pontine stroke who developed PLC and those who did not. Additional variables were statistically tested to determine any differences between the two groups.

### 2.2 Participants

Medical records were obtained from patients diagnosed with first-ever solitary pontine stroke and admitted to a tertiary inpatient rehabilitation hospital between January 1, 2011, and December 31, 2021. Data were reviewed using the clinical data retrieval system. Among these patients, those who underwent ^18^F-FDG-PET within 14 days of admission were included in further studies.

The inclusion criteria were as follows: (1) had a hemorrhagic or ischemic stroke involving the pons confirmed by brain computed tomography (CT) or MRI; (2) underwent ^18^F-FDG-PET imaging at our institution within 6 months of stroke onset; and (3) aged 20 years or older at the time of stroke.

The exclusion criteria were as follows: (1) cognitive impairment, defined as a Mini-Mental State Examination (MMSE) score of less than 23; (2) pontine lesions extending to adjacent brain structures or accompanied by SAH or IVH; (3) presence of old cerebral lesions larger than 3 mm in diameter on MRI; (4) diagnosis of brain lesions other than stroke, such as traumatic or hypoxic brain injury; (5) diagnosis of neurodegenerative disorders, such as Parkinson’s disease; and (6) a history of any underlying mood or anxiety disorders.

A total of 89 patients initially met the inclusion criteria. Of these, 23 were excluded due to MMSE scores below 23; 6 were excluded due to lesions extending beyond the pons; 9 were excluded due to old cerebral lesions larger than 3 mm; 2 were excluded due to a documented history of depression before stroke onset; no patients were excluded due to traumatic or hypoxic brain injury or neurodegenerative disease. Ultimately, 49 patients were included in the final analysis.

Patients were classified into two groups based on the presence or absence of newly developed PLC following stroke. The experimental group (PLC group) consisted of patients who met all inclusion criteria and exhibited new-onset PLC after stroke. The control group (non-PLC group) included patients who met all inclusion criteria but did not develop PLC following stroke.

PLC diagnosis was based on medical record review during hospitalization using the following criteria: (1) new-onset episodes of sudden, uncontrollable emotional expression; (2) emotional response more intense compared with the triggering stimulus; and (3) emotional expression not reflective of the patient’s underlying mood. PLC severity was assessed using the PLACS (2, 21).

### 2.3 Data collection and variables

Demographic information, including age at onset, duration since onset, and sex, was obtained from electronic medical records. Clinical data included assessments, such as the National Institutes of Health Stroke Scale, Fugl–Meyer Assessment (motor/sensory scales), MMSE, Geriatric Depression Scale, and Medication Quantification Scale.

Only data from patients who underwent brain ^18^F-FDG-PET CT at our institution were included in the analysis. Additional clinical information collected included medication history, cognitive function, and depression severity during hospitalization. Stroke diagnosis was confirmed via neurological examinations and imaging studies (CT or MRI) performed at the time of onset, as determined by the attending physician.

Age and stroke lesion volume, previously identified as factors influencing metabolism on ^18^F-FDG-PET in previous studies, were evaluated and used as covariates in the statistical analysis. MRI analysis provided information on lesion size and stroke type, while ^18^F-FDG-PET imaging was used to assess brain metabolism.

### 2.4 Imaging acquisition and analysis

^18^F-FDG-PET images were acquired within 14 days of admission using a GE Discovery 600 PET/CT scanner (GE Medical Systems, Milwaukee, WI, USA). The images included low-resolution CT scans and high-resolution three-dimensional PET images (4.8-mm full width) with attenuation-corrected emission data reconstructed in a 128 × 128 × 35 matrix. The pixel size was 1.95 × 1.95 × 4.25 mm, using a transaxial 8.5-mm Hanning filter and an 8.5-mm axial ramp filter.

Medical imaging specialists converted the images from Digital Imaging and Communications in Medicine files to the Neuroimaging Informatics Technology Initiative format. The ^18^F-FDG-PET images were realigned and normalized using a standard PET template from the Montreal Neurological Institute. Subsequently, each image was smoothed using a three-dimensional Gaussian filter (8 mm^3^ full width at half maximum). All image processing and analyses were performed using the SPM software version 12^1^ implemented in MATLAB R2018a.

Brain metabolism was compared between the PLC and non-PLC groups using voxel-wise two-sample t-tests in SPM to identify brain regions with statistically significant differences. To correct for multiple comparisons, clusters were reported with a threshold of *p* < 0.05 (family-wise error-corrected), and a cluster size threshold of 10 voxels was used to determine statistical significance. Age and total lesion volume were adjusted as covariates. Multiple regression analysis was conducted using age and stroke lesion volume as covariates, with a threshold of uncorrected *p* < 0.001, to identify the brain area that was significantly correlated with PLC severity, as measured using PLACS, within the PLC group. Anatomical localization was determined using an Automated Anatomical Labeling program.^2^

### 2.5 Statistical analysis

Demographic and clinical data, including age, sex, stroke type, duration since onset, lesion volume, National Institutes of Health Stroke Scale, Korean version of the Modified Barthel Index, MMSE, and Geriatric Depression Scale, were obtained. Statistical analyses were performed using SPSS Statistics software (version 25.0; IBM, Armonk, NY, USA).

Lesion volumes were measured from CT scans using the ABC/2 formula (22, 23). The normality of continuous variables was assessed using the Kolmogorov–Smirnov test. Age, lesion, volume, National Institutes of Health Stroke Scale, Modified Barthel Index, MMSE, Geriatric Depression Scale, and PLACS scores followed a normal distribution, while the duration since onset did not. Independent t-tests were used for parametric comparisons, and the Mann–Whitney U test was used for nonparametric variables. Fisher’s exact test was applied to categorical variables (sex and stroke type). A *p* < 0.05 was considered statistically significant.

## 3 Results

### 3.1 Participants characteristics

Overall, 49 patients with pontine stroke were included in the study, comprising the PLC (*n* = 20) and the non-PLC groups (*n* = 29). Among them, 33 had hemorrhagic strokes and 16 had ischemic strokes. The PLC group had slightly younger participants and a higher proportion of female patients than did the non-PLC group; however, these differences were not statistically significant. Other demographic characteristics also showed no significant differences between the two groups (Table 1).

**Table 1:** Demographic characteristics of patients with pontine stroke.

Variable	Control group (*n* = 29)	PLC group (*n* = 20)	*p*
Age, years	51.3 ± 13.0	45.9 ± 11.3	0.107
Sex (male/female)	23/6	14/6	0.512
Duration since onset, days	65.0 (66.0)	79.0 (110.3)	0.371
Stroke type (ischemic/hemorrhagic)	9/20	7/13	1.000
Lesion volume, mL	6.3 ± 4.7	6.6 ± 4.6	0.803
NIHSS (0–42)	12.9 ± 10.1	12.3 ± 5.1	0.109
MBI (0–100)	30.4 ± 24.9	34.4 ± 24.6	0.668
MMSE (0–30)	27.8 ± 2.4	28.1 ± 2.3	0.621
GDS (0–30)	13.0 ± 7.5	15.1 ± 9.3	0.136
PLACS Total (0–54)	0 ± 0	14.1 ± 11.1	<0.001 *

Values are presented as means ± standard deviations for normally distributed continuous variables and as medians (interquartile ranges) for non-normally distributed variables. PLC, pathological laughing and crying; NIHSS, National Institutes of Health Stroke Scale; MBI, Modified Barthel Index; MMSE, Mini-Mental State Examination; GDS, Geriatric Depression Scale; PLACS, Pathological Laughter and Crying Scale; *p < 0.05.

### 3.2 Metabolic correlates of pathological laughing and crying

**Figure 1:**
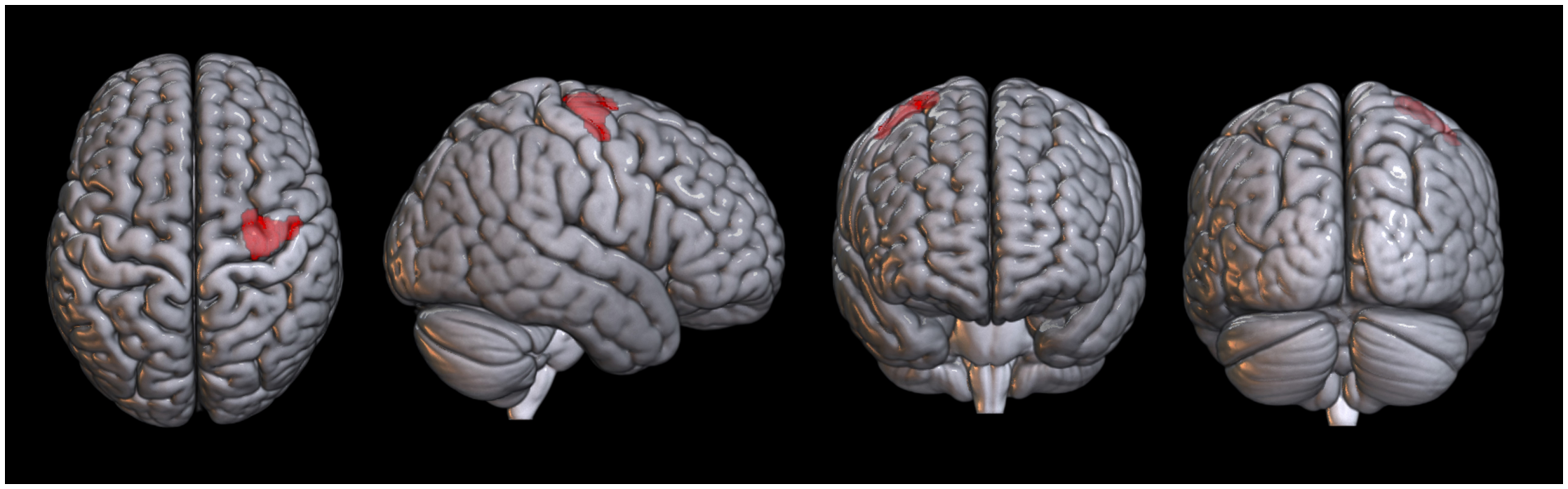
Spatial distributions of decreased glucose metabolism in the pathological laughing and crying group following pontine stroke compared to the control group (*P*_family-wise error-corrected_ < 0.05, *k* = 10).

**Figure 2:**
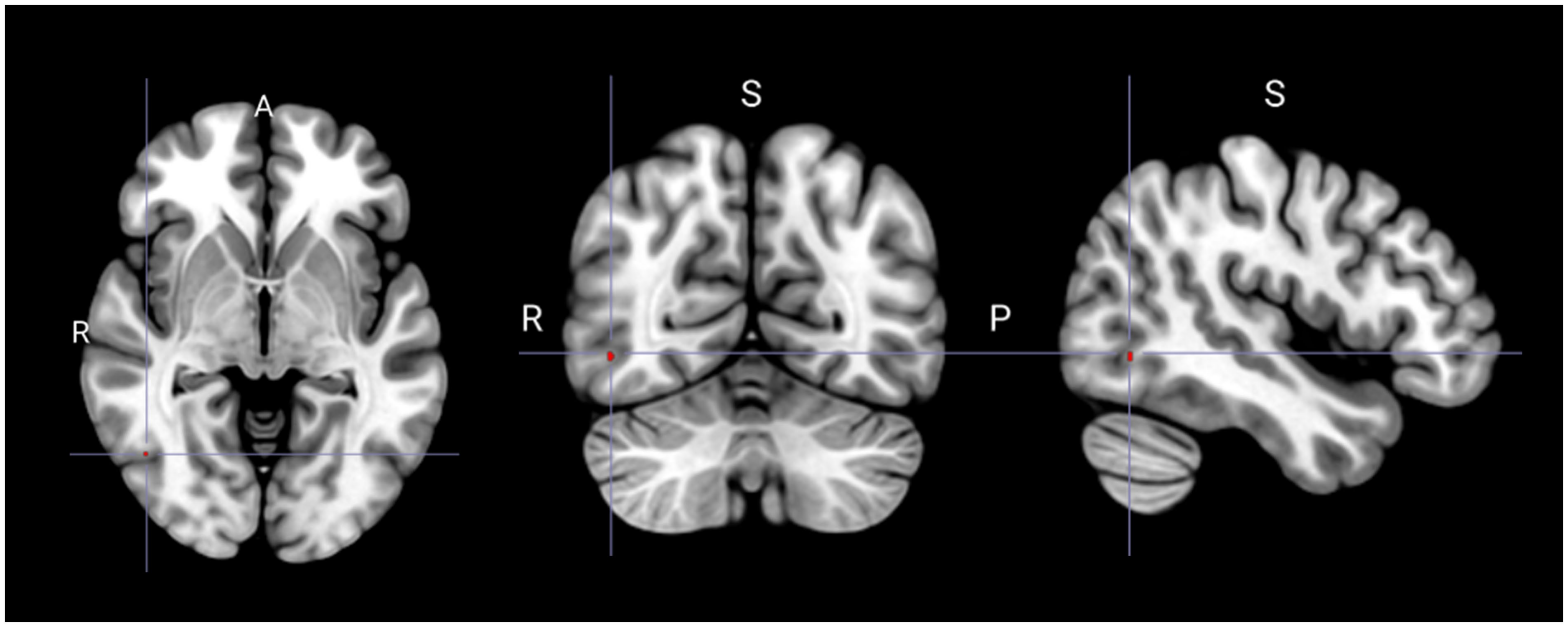
Spatial distribution of reduced glucose metabolism correlated with increased severity of pathological laughing and crying (*P*_uncorrected_ < 0.001). S, superior; R, right; P, posterior.

Compared with that in the non-PLC group, the PLC group exhibited significant hypometabolism in the right superior frontal gyrus (Figure 1; Table 2). Multiple regression analysis revealed that decreased metabolism in the right inferior or middle temporal gyri correlated with a higher PLACS score, indicating greater PLC severity (Figure 2; Table 3). No brain region showed a positive correlation.

**Table 2:** Brain regions with altered glucose metabolism in patients with pathological laughing and crying following pontine stroke.

Metabolism	Area	Coordinate			*t* Score	*z* Score	Cluster
		x	y	z			
Decreased	Right superior frontal gyrus	28	−6	70	5.41	4.72	250

*P*_family-wise error-corrected_ < 0.05, *k* = 10.

**Table 3:** Brain regions showing decreased glucose metabolism correlated with higher pathological laughter and crying scale scores.

Metabolism	Area composition	Coordinate			*t* Score	*z* Score	Cluster
		x	y	z			
Decreased	Right inferior temporal gyrus Right middle temporal gyrus	46	−64	−4	3.65	3.11	2

*P*_uncorrected_ < 0.001.

## 4 Discussion

Despite extensive research on PLC etiology, its exact pathogenesis remains unclear. While structural and functional MRI studies have highlighted the significance of pontine lesions, no study has assessed the functional cerebral metabolic alterations in patients with pontine stroke using ^18^F-FDG-PET imaging. To our knowledge, this is the first study where glucose metabolic changes associated with PLC development have been investigated.

PLC is traditionally believed to result from cortical dysregulation of the upper brainstem regions, potentially due to structural or functional damage to neurotransmitter neurons or their associated pathways (1). Some researchers have suggested that PLC arises from a distributed network involving the frontal cortex, brainstem, and cortico-ponto-cerebellar pathways (10), which play a role in modulating contextually appropriate emotional responses. Additionally, partial cerebellar deafferentation has been proposed as a contributing factor (3, 24). Previous studies have been conducted to analyze gray or white matter abnormalities, as well as decreased fractional anisotropy, to explain the anatomical basis of PLC. MRI-based tractography studies have also been conducted to demonstrate the existence of related networks (16).

Furthermore, monoaminergic neurotransmitter systems, particularly serotonin, have been implicated in both mood regulation and affective expression, supporting the hypothesis that serotonergic dysfunction may contribute to PLC following stroke. PLC, especially crying episodes, has shown a strong response to serotonergic agents, with selective serotonin reuptake inhibitors recommended as first-line pharmacotherapy for PLC (25). The brainstem, particularly the pons, has also been implicated in emotional lability (12). As highlighted in various studies, the pons appear to be a key component of the PLC network, with the rostral basis pontis showing the strongest association with PLC (3, 26).

Lesion network-symptom mapping studies using resting-state functional MRI have identified alterations in default mode, sensorimotor, affective, and cerebellar networks in patients with post-stroke PLC (8). Similarly, PLC in amyotrophic lateral sclerosis has been associated with decreased functional connectivity in cognitive networks, including default mode, frontoparietal, salience, and sensory-motor networks (17). Another study revealed that PLC-related lesions exhibited positive connectivity with the cingulate and temporomesial cortices, striatum, hypothalamus, mesencephalon, and pons while showing negative connectivity with the primary motor and sensory cortices. Moreover, these regulatory pathways often involve the frontal and temporal lobes (11).

In our study, we found that the superior frontal, middle temporal, and inferior temporal gyri, which are all components of the default mode network (DMN), showed significant metabolic alterations in patients with PLC. As many previously mentioned, functional imaging studies have shown the involvement of the DMN in PLC, we hypothesized that the DMN may play a role in PLC development. While functional MRI connectivity analyses or more conventional gray and white matter evaluations were primarily used in these studies, in our study, we identified a glucose metabolic functional connection between the DMN and PLC.

The DMN is predominantly active during the resting state and decreases in activity as individuals engage in tasks with external demands (27). It has been proposed to play a key role in social cognition, including empathic responses and affective processing (28). The findings from functional MRI studies have suggested that the pathology within the DMN is closely associated with emotion-related processes, particularly those crucial for social cognition, cognitive-emotional regulation, and emotional processing (29–31). Moreover, altered DMN activity has been linked to depression and anxiety (32, 33). Based on these insights, our findings suggest that PLC may arise from dysregulated activity within the DMN, leading to impaired modulation of emotional responses.

Supporting this hypothesis, a previous brain single-photon emission computed tomography study on a patient with pontine ischemic stroke exhibiting pathological laughing revealed marked hypoperfusion in the right inferior frontal and temporoinsular lesions, suggesting the presence of diaschisis (34). This finding is partially compatible with the hypometabolic regions identified in our study, further supporting the possibility that diaschisis may contribute to PLC development. Therefore, we suggest that pontine stroke lesions may induce diaschisis, resulting in hypometabolic changes within the DMN, which ultimately contribute to PLC development.

Connections between the prefrontal cortex and brainstem nuclei are believed to play a crucial role in the regulation of emotional expression, reinforcing the findings of this study. The prefrontal cortex, in coordination with paralimbic regions, forms a network that controls emotional expression, which is further modulated by the cerebellum via pathways through the basis pontis (10). Notably, cortico-pontine fibers involved in spontaneous emotional expressions originate from the prefrontal cortex and descend through the internal capsule, thalamus, and brain stem. These pathways are distinct from those controlling voluntary motor functions, highlighting a separate neural substrate for involuntary affective responses (35).

In our study, we did not fully elucidate all aspects of PLC pathophysiology; however, we provided valuable insights into its neural correlates. Traditionally, PLC has been interpreted through circuit-based frameworks, top-down versus bottom-up regulatory models, or by attributing it to a single region. In contrast, our findings suggest that PLC may result from dysregulation across distinct functionally related regions, which may or may not be part of well-defined neural circuits. These identified regions are involved in emotional expression, and their disinhibition may contribute to PLC manifestation. Our results contribute to a clearer understanding of PLC-associated brain regions and may provide a basis for future analyses of PLC pathophysiology.

### 4.1 Limitations

The relatively small sample size is a limitation of this study. Additionally, the number of voxels significantly associated with PLC severity, as measured using the PLACS, was limited. In this study, we also exclusively focused on patients with pontine stroke. Furthermore, electroencephalography was not performed, making it difficult to rule out seizure activity. While PLC may resemble gelastic or dacrystic seizures (forms of complex partial seizures), electroencephalography would have been helpful in differentiating PLC from seizure-related manifestations (36). As our findings suggest the involvement of distinct brain areas in PLC, it would be valuable to investigate patients with stroke with lesions outside the pons and incorporate a broader range of diagnostic tools. A larger sample size and patients with stroke with lesions in other brain regions should be included in future research to validate these associations.

### 4.2 Conclusion

Our findings suggest that metabolic alterations in remote brain regions may contribute to PLC development. Rather than being confined to a single brain region, PLC involves multiple interconnected areas that may play roles in emotional expression and impulse control. Further research is needed to explore the complex interactions among multiple brain regions in PLC and identify therapeutic targets within these networks.

## Data Availability

The raw data supporting the conclusions of this article will be made available by the authors without undue reservation.

## Footnotes

^1^ http://www.fil.ion.ucl.ac.uk/spm/

^2^ https://www.gin.cnrs.fr/AAL/
